# A Filamentous Bacteriophage Protein Inhibits Type IV Pili To Prevent Superinfection of Pseudomonas aeruginosa

**DOI:** 10.1128/mbio.02441-21

**Published:** 2022-01-18

**Authors:** Amelia K. Schmidt, Alexa D. Fitzpatrick, Caleb M. Schwartzkopf, Dominick R. Faith, Laura K. Jennings, Alison Coluccio, Devin J. Hunt, Lia A. Michaels, Aviv Hargil, Qingquan Chen, Paul L. Bollyky, David W. Dorward, Jenny Wachter, Patricia A. Rosa, Karen L. Maxwell, Patrick R. Secor

**Affiliations:** a Division of Biological Sciences, University of Montana, Missoula, Montana, USA; b Department of Biochemistry, University of Toronto, Toronto, Ontario, Canada; c Division of Infectious Diseases and Geographic Medicine, Department of Medicine, Stanford University, Stanford, California, USA; d Research Technologies Branch, National Institute of Allergy and Infectious Diseases, National Institutes of Health, Bethesda, Maryland, USA; e Laboratory of Bacteriology, Rocky Mountain Laboratories, National Institute of Allergy and Infectious Diseases, National Institutes of Health, Bethesda, Maryland, USA; University of Pittsburgh School of Medicine

**Keywords:** Pf4, PilC, *Pseudomonas aeruginosa*, filamentous bacteriophage, superinfection exclusion, twitching motility, type IV pili

## Abstract

Pseudomonas aeruginosa is an opportunistic pathogen that causes infections in a variety of settings. Many P. aeruginosa isolates are infected by filamentous Pf bacteriophage integrated into the bacterial chromosome as a prophage. Pf virions can be produced without lysing P. aeruginosa. However, cell lysis can occur during superinfection, which occurs when Pf virions successfully infect a host lysogenized by a Pf prophage. Temperate phages typically encode superinfection exclusion mechanisms to prevent host lysis by virions of the same or similar species. In this study, we sought to elucidate the superinfection exclusion mechanism of Pf phage. Initially, we observed that P. aeruginosa that survive Pf superinfection are transiently resistant to Pf-induced plaquing and are deficient in twitching motility, which is mediated by type IV pili (T4P). Pf utilize T4P as a cell surface receptor, suggesting that T4P are suppressed in bacteria that survive superinfection. We tested the hypothesis that a Pf-encoded protein suppresses T4P to mediate superinfection exclusion by expressing Pf proteins in P. aeruginosa and measuring plaquing and twitching motility. We found that the Pf protein PA0721, which we termed Pf superinfection exclusion (PfsE), promoted resistance to Pf infection and suppressed twitching motility by binding the T4P protein PilC. Because T4P play key roles in biofilm formation and virulence, the ability of Pf phage to modulate T4P via PfsE has implications in the ability of P. aeruginosa to persist at sites of infection.

## INTRODUCTION

Pseudomonas aeruginosa is an opportunistic pathogen that causes infection in wounds, on medical hardware, and in the airways of people with cystic fibrosis. P. aeruginosa itself can be infected by a variety of bacteriophages (phages). For example, many P. aeruginosa isolates are infected by temperate filamentous Pf phage, which can integrate into the bacterial chromosome as a prophage (1, 2). During P. aeruginosa growth as a biofilm or at sites of infection, the Pf prophage is induced, and filamentous virions are produced (3–6). Like other filamentous phages, Pf can be extruded from the host cell without killing the host, allowing Pf virions to accumulate to high titers in biofilms (10^11^/mL) (7) and infected tissues (10^7^/gram) (8). However, cell lysis can occur when Pf superinfects P. aeruginosa, which occurs when multiple virions infect the same cell or when superinfective Pf variants emerge that contain mutations in the phage *c* repressor gene *Pf4r* (7, 9). Pf4-mediated cell lysis contributes to the maturation and dispersal stages of the P. aeruginosa biofilm life cycle (10–13).

Temperate phages typically encode superinfection exclusion mechanisms to stave off infection by competing phages in the environment. A common theme among superinfection exclusion mechanisms are proteins that inhibit or modify phage cell surface receptors such as type IV pili (T4P) (14), a common cell surface receptor for phages, including Pf4 (15). Many P. aeruginosa phages encode proteins that inhibit T4P to prevent superinfection (16). Specific examples include the Aqs1 protein encoded by phage DMS3 and the Tip protein encoded by phage D3112, which both inhibit T4P by binding to the T4P ATPase PilB (17, 18). Pf4 uses T4P as a cell surface receptor (15); however, a superinfection exclusion mechanism has not been characterized for the Pf phages that reside in P. aeruginosa genomes.

In this study, we show that the smallest protein encoded by Pf4, which we call PfsE (Pf superinfection exclusion), transiently inhibits T4P assembly through an interaction with the T4P platform protein PilC, providing resistance to further infection by T4P-dependent phages. By introducing point mutations to PfsE, we identified two aromatic residues (Y16 and W20) that may be required for PilC binding, T4P inhibition, and resistance to T4P-dependent phages. Furthermore, phage Pf4 engineered to lack the *pfsE* gene is able to kill P. aeruginosa more efficiently than the wild-type phage, demonstrating that this mechanism of superinfection reduces P. aeruginosa cell lysis. Filamentous inoviruses such as Pf are widespread among bacterial genomes, with even a few examples infecting Archaea (19). Thus, the superinfection exclusion mechanism described here may be relevant to many species of filamentous phage that infect diverse bacterial hosts.

## RESULTS

### Type IV pili are transiently suppressed in response to Pf4 superinfection.

**FIG 1:**
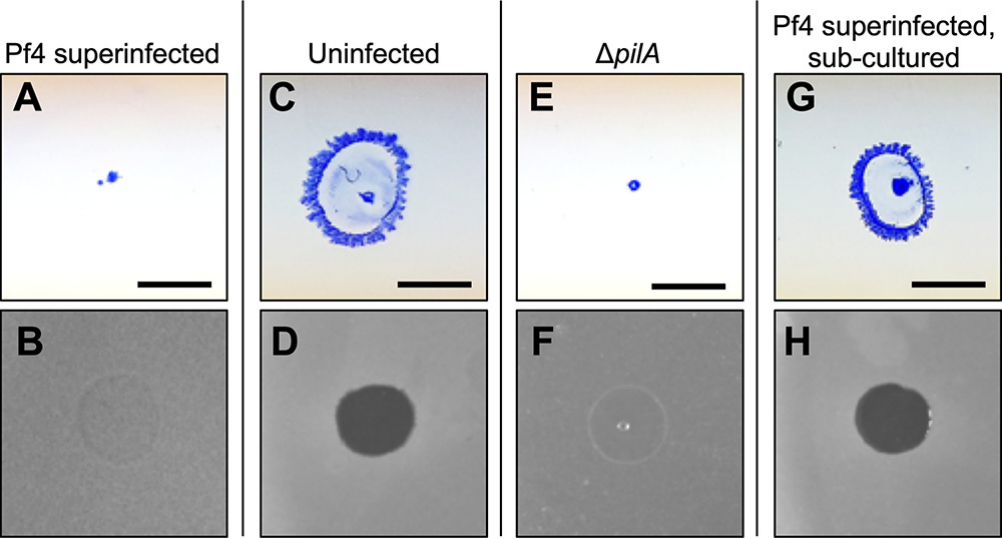
Pf4 superinfection transiently suppresses twitching motility and promotes resistance to Pf4-induced plaquing. Twitch assays were performed by stabbing the indicated strain through the agar on a petri dish to the plastic surface below. After 24 h, the agar was removed and bacteria on the plastic dish were stained with Coomassie (upper panels A, C, E, and G). To measure sensitivity of P. aeruginosa to Pf4 superinfection, lawns of the indicated strains were spotted with 10^6^ PFU of Pf4 in 3 μL (lower panels B, D, F, and H). Strains tested include (A and B) PAO1 superinfected with Pf4, (C and D) uninfected PAO1, (E and F) the twitch-deficient type IV pili mutant Δ*pilA*, and (G and H) Pf4 superinfected PAO1 that was subcultured in phage-free broth and replated. Scale bar 5 mm.

While working with phage Pf4 we noticed an interesting phenomenon where the surviving cells in a culture of PAO1 superinfected with Pf4 showed a decrease in twitching motility (Fig. 1A). As Pf4 uses T4P as a cell surface receptor (15), we tested the ability of these nontwitching cells to mediate resistance to Pf4-induced plaquing. We found that these cells were highly resistant to lysis by Pf4 virions (Fig. 1B) compared to uninfected PAO1, which retain the ability to twitch (Fig. 1C) and are sensitive to Pf4 superinfection (Fig. 1D). The resistance observed was similar to that seen for a PAO1 Δ*pilA* mutant, which completely lacks pilus on the cell surface (Fig. 1E and F). To determine if Pf4 superinfection selected for T4P-null mutants or transiently suppressed T4P expression, bacteria collected from Pf4-resistant lawns were subcultured in phage-free growth medium for 18 h, and then their ability to twitch and sensitivity to Pf4-induced plaquing was tested. Twitching motility and sensitivity to Pf4 superinfection was restored in subcultured bacteria (Fig. 1G and H), indicating that heritable mutations in T4P genes were not responsible for the twitch-deficient and Pf4-resistance phenotypes.

### PfsE suppresses twitching motility and protects P. aeruginosa from Pf4 superinfection.

**FIG 2:**
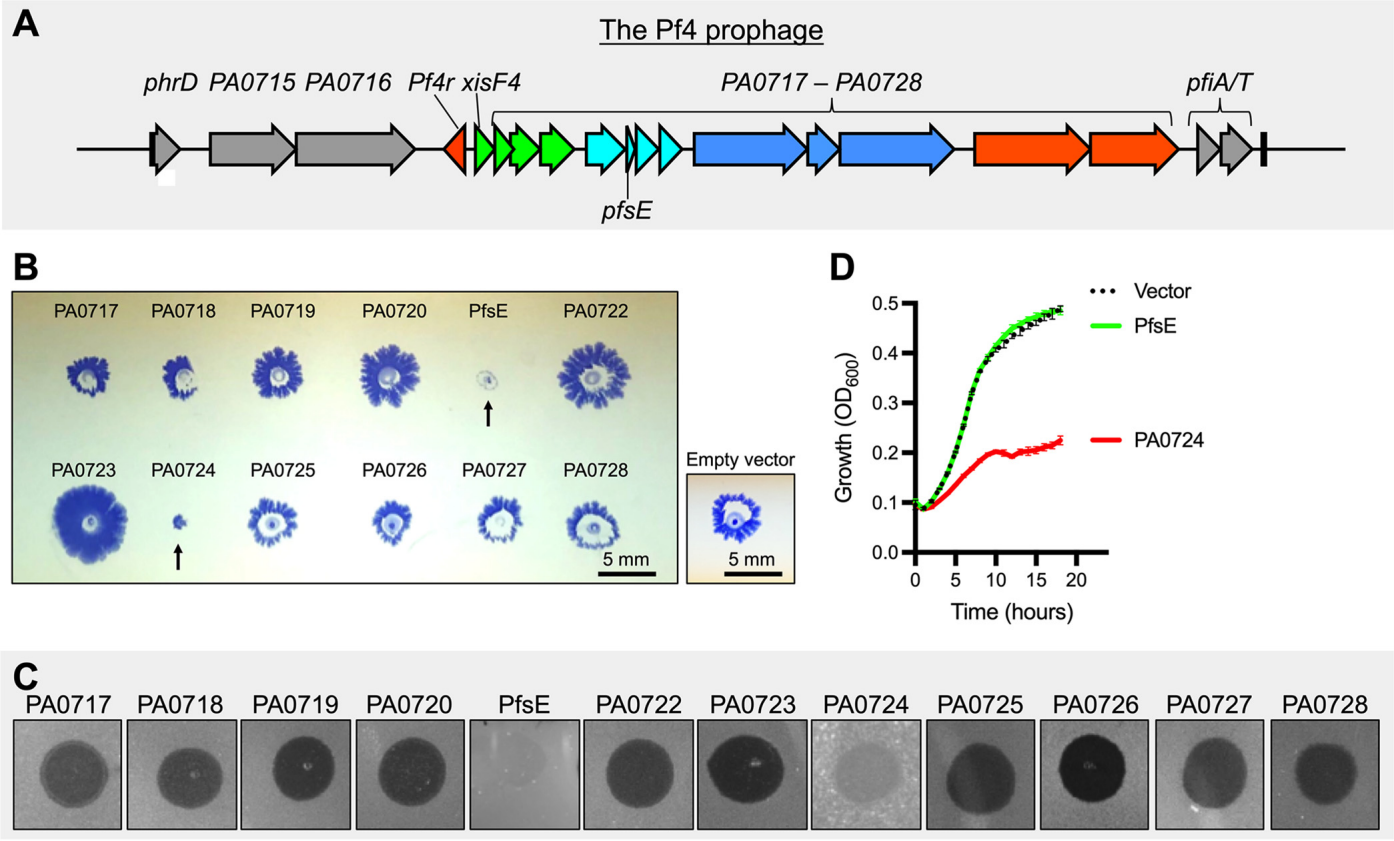
PfsE suppresses twitching motility and protects P. aeruginosa from Pf4 superinfection. (A) The Pf4 prophage is composed of core genes that are essential for the phage to complete its life cycle (*Pf4r* to *PA0728*) and flanking moron regions (gray) that add “more on” to the core genome (54). (B) Genes *PA0717*–*PA0728* in the core Pf4 genome were placed under the control of an arabinose-inducible promoter and expressed individually in P. aeruginosa PAO1^ΔPf4^. Twitching was measured in the indicated strains after 24 h; arrows indicate strains with reduced twitching motility. Bar 5 mm. (C) 10^6^ PFU of Pf4 were spotted onto lawns of PAO1^ΔPf4^ expressing the indicated phage protein. (D) Growth curves in liquid culture for PAO1^ΔPf4^ bacteria carrying the indicated expression vector. Cultures were grown in LB supplemented with 0.1% arabinose. Results are the mean ± SEM of three experiments.

Many temperate phages possess superinfection exclusion mechanisms that prevent re-infection of an already infected cell (20). We hypothesized that a Pf4-encoded protein would suppress T4P as a mechanism to prevent Pf4 superinfection and lysis of the host cell. To test this hypothesis, we first deleted the Pf4 prophage from our in-house PAO1 strain (PAO1^ΔPf4^). Pf4 proteins encoded by *PA0717-PA0728* in the core Pf4 genome (Fig. 2A) were then expressed individually from a plasmid in PAO1^ΔPf4^, and twitching motility and sensitivity to Pf4-mediated lysis were then assessed. We identified two proteins, PA0721 and PA0724, that suppressed twitching motility when overexpressed (Fig. 2B) and promoted resistance to Pf4 plaquing (Fig. 2C). PA0721 is a small 30 residue uncharacterized protein, and PA0724 is the Pf4 minor coat protein CoaA, which is involved in receptor binding during the initial steps of infection (2). These results were consistent with a previous study that found these proteins promote resistance to T4P-dependent long-tailed dsDNA phages DMS3m and JBD30 (19).

To determine if the observed twitching inhibition and phage resistance was a direct result of the biological function of these proteins or was due to toxicity of the overexpressed proteins, we examined the growth rates of cells expressing these proteins. We found that PAO1^ΔPf4^ expressing PA0724 grew poorly compared to cells expressing PA0721 or PAO1^ΔPf4^ carrying an empty expression vector (Fig. 2D). These observations suggest that PA0724 expression is toxic to P. aeruginosa, and it is possible that the twitch-deficient and phage resistance phenotypes associated with PA0724 expression are a result of this toxicity rather than a specific superinfection exclusion mechanism. Therefore, we turned our attention toward characterizing PA0721, which we refer to herein as PfsE (Pf superinfection exclusion).

### T4P are not apparent on cells expressing PfsE.

**FIG 3:**
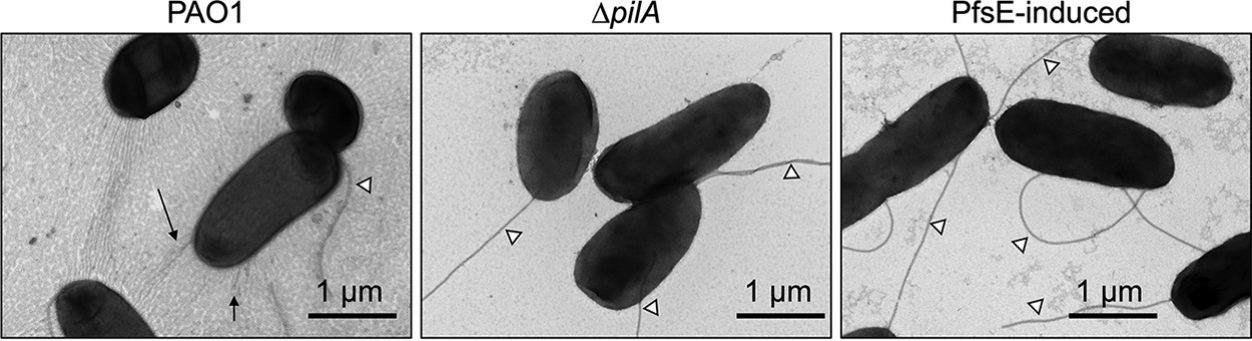
Type IV pili are not apparent on cells expressing PfsE. Transmission electron microscopy of the indicated strains of P. aeruginosa was performed. Midlogarithmic cells were washed, fixed, placed on a grid, and negatively stained with uranyl acetate. Arrows indicate potential pili on PAO1 cells. White triangles indicate flagella.

Twitching motility requires bacteria to extend their pili outwards from the cell surface and then retract them to move along a solid surface. Thus, PfsE could inhibit twitching motility by preventing either pilus retraction or extension. If PfsE inhibits T4P retraction, cells are anticipated to have a piliated or hyperpiliated morphology. If PfsE inhibits T4P extension, then cells are expected to have few or no pili. To determine if PfsE interferes with extension or retraction, we used transmission electron microscopy to look for the presence of pili on the cell surface. We found that cells expressing PfsE showed no visible pili on the surface (Fig. 3), while wild-type PAO1 cells had structures consistent with T4P (Fig. 3). The lack of pili observed on the cells expressing PsfE was similar to a PAO1 Δ*pilA* mutant (Fig. 3), which is known to completely lack surface piliation (21). These data suggest that PfsE inhibits T4P extension rather than retraction.

### PfsE protects P. aeruginosa from other T4P-dependent phage species.

**FIG 4:**
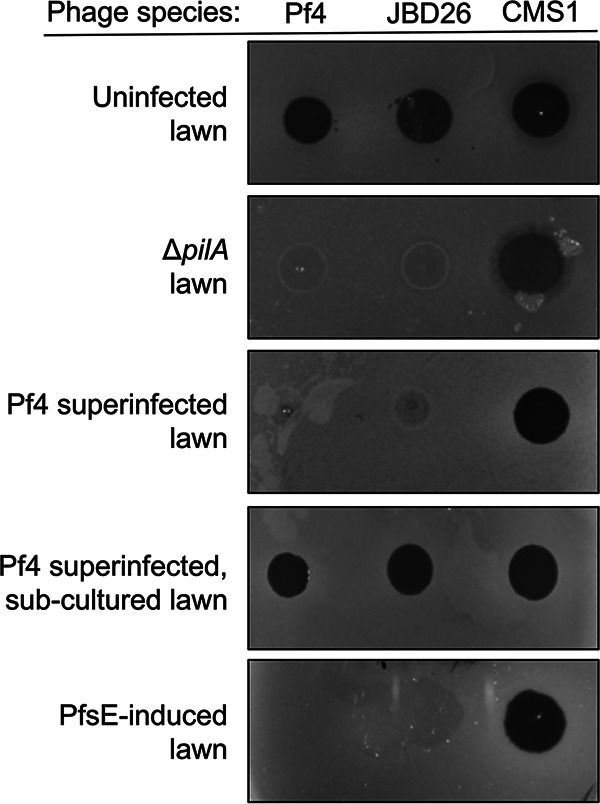
Pf4 superinfection and expression of PfsE promotes resistance to type IV pili (T4P)-dependent bacteriophages. Representative images of P. aeruginosa PAO1 lawns spotted with 10^6^ PFU of Pf4, JBD26 (both T4P-dependent phages), or CMS1 (a T4P-independent phage). See also Fig. S1.

Many phages use the T4P as a cell surface receptor to infect bacteria (14). We hypothesized that the transient T4P suppression by PfsE that protected against Pf4 superinfection would also protect P. aeruginosa from nonfilamentous phages. To test this hypothesis, we examined the ability of phage JBD26, a temperate long-tailed dsDNA phage that uses the pilus as a cell surface receptor, to form plaques on lawns of PAO1, PAO1 Δ*pilA*, PAO1 superinfected with Pf4, PAO1 superinfected with Pf4 and then subcultured in phage-free media, or PAO1 expressing PfsE from a plasmid. Like Pf4, JBD26 was not able to infect cells that were superinfected by Pf4 or cells expressing PfsE (Fig. 4). We also tested the ability of phage CMS1, which does not depend on the pilus for infection, to form plaques on these strains. As expected, the plaquing ability of these phages was not affected by the absence of T4P (Δ*pilA*), expression of PsfE, or superinfection by Pf4 (Fig. 4). Furthermore, Pf4 superinfection and expression of PfsE were able to prevent infection by phage OMKO, a lytic pili-dependent phage, but not LPS-5, another pili-independent phage species (Fig. S1 in the supplemental material). These results demonstrate that PfsE protects P. aeruginosa from T4P-dependent phage species.

10.1128/mBio.02441-21.1 FIG S1 Pf4 superinfection and expression of PfsE promotes resistance to type IV pili (T4P)-dependent lytic bacteriophages. Representative images of P. aeruginosa PAO1 lawns spotted with 10^6^ PFUs OMKO (T4P-dependent lytic phage), or LPS-5 (a T4P-independent lytic phage). Note plaques are visualized as bright spots in these images. Download FIG S1, TIF file, 0.8 MB. Copyright © 2022 Schmidt et al. 2022 Schmidt et al. https://creativecommons.org/licenses/by/4.0/ This content is distributed under the terms of the Creative Commons Attribution 4.0 International license.

We also tested the ability of PfsE to inhibit flagellum-dependent swimming motility. PAO1^ΔPf4^ expressing wild-type PfsE or PfsE point mutants did not affect swimming motility compared to PAO1^ΔPf4^ carrying an empty expression vector (Fig. S2), indicating that PfsE does not affect flagellum-dependent swimming motility, which is consistent with our previous observation that Pf4 superinfection does not affect swimming motility in P. aeruginosa PAO1 (5).

10.1128/mBio.02441-21.2 FIG S2 PfsE expression does not affect swimming motility in P. aeruginosa. Swimming motility of P. aeruginosa growing on swim agar was measured in PAO1^ΔPf4^ expressing wild-type or modified PfsE or an empty expression vector (pHERD20T). Results are mean ± SEM of 6 experiments, unpaired Student’s *t* test. Download FIG S2, TIF file, 0.2 MB. Copyright © 2022 Schmidt et al. 2022 Schmidt et al. https://creativecommons.org/licenses/by/4.0/ This content is distributed under the terms of the Creative Commons Attribution 4.0 International license.

### Deletion of *pfsE* increases Pf4 virulence against P. aeruginosa.

To definitively show that PsfE expression from Pf phage provides resistance to superinfection, we attempted to delete the *pfsE* gene from Pf4. All attempts to delete *pfsE* from the Pf4 prophage integrated into the PAO1 chromosome failed. We hypothesized that inactivating *pfsE* resulted in unregulated replication of Pf4, killing *pfsE* mutants, similar to how Pf4 kills P. aeruginosa PAO1 when the global repressors *mvaT* and MvaU are both disabled (15). To test this hypothesis, we attempted to delete *pfsE* from the PAO1 Δ*intF4* background. IntF4 (PA0728) is a Pf4-encoded site-specific tyrosine recombinase that catalyzes Pf4 prophage integration and excision (8, 9). In Δ*intF4*, the Pf4 prophage is trapped in the chromosome, preventing infectious virions from being produced (8, 15). We were successful in deleting *pfsE* from Δ*intF4*, creating the double mutant Δ*intF4/pfsE*, suggesting that when *pfsE* is inactivated, Pf4 replication kills P. aeruginosa.

**FIG 5:**
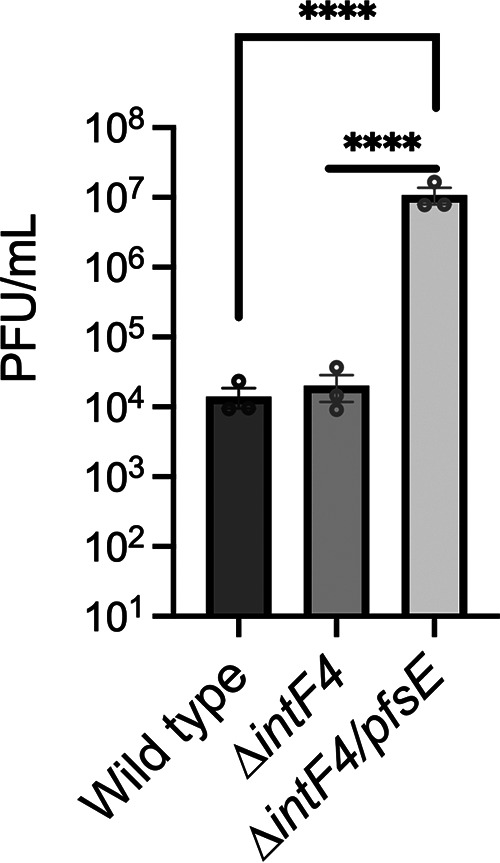
Pf4 virions not encoding *pfsE* are more virulent against P. aeruginosa. Wild-type and mutant Pf4 virions were induced by expressing both the Pf4 excisionase (*xisF4*) and integrase (*intF4*) in *trans* in wild-type, Δ*intF4*, or Δ*intF4/pfsE* backgrounds. Note that deletion of *pfsE* alone from the Pf4 prophage was not possible. Wild-type, Δ*intF4*, and Δ*intF4/pfsE* Pf4 virion titers were measured by qPCR and normalized to 6.95 × 10^7^ virions per mL. Virions were then spotted as a 10× dilution series on lawns of ΔPf4 to enumerate PFU. Results are mean ± SEM of three experiments, unpaired Student's *t* test, ****, *P* < 0.0001.

We hypothesized that Δ*intF4/pfsE* Pf4 virions that lack the *pfsE* gene would not be able to regulate superinfection of the host bacterium, increasing host cell lysis. To test this, we induced and collected Pf4 virions from wild-type, Δ*intF4*, and Δ*intF4/pfsE* P. aeruginosa. To induce Pf4 virions from these strains, the Pf4 excisionase XisF4 was expressed in *trans* from a plasmid under the control of an arabinose-inducible promoter (9). To complement the *ΔintF4* mutation, IntF4 was also expressed from a plasmid in all strains tested. After overnight growth (18 h) in lysogeny broth (LB) supplemented with 0.1% arabinose, bacterial supernatants were filtered and DNase-treated, and Pf4 titers measured by qPCR, as previously described (22). Phage titers in each supernatant (wild type, Δ*intF4*, and Δ*intF4/pfsE*) were normalized to 6.95 × 10^7^ virions per mL and were plated on a lawn of PAO1^ΔPf4^ to determine how infective the mutant phages were. While the Δ*intF4* mutant phage titer was equal to wild-type Pf4, the Δ*intF4/pfsE* mutant phage was approximately 1,000-fold more infective (Fig. 5), indicating that *pfsE* restricts Pf4 infection and thereby protects the bacterial host from lysis.

### PfsE binds to the T4P inner-membrane protein PilC.

**FIG 6:**
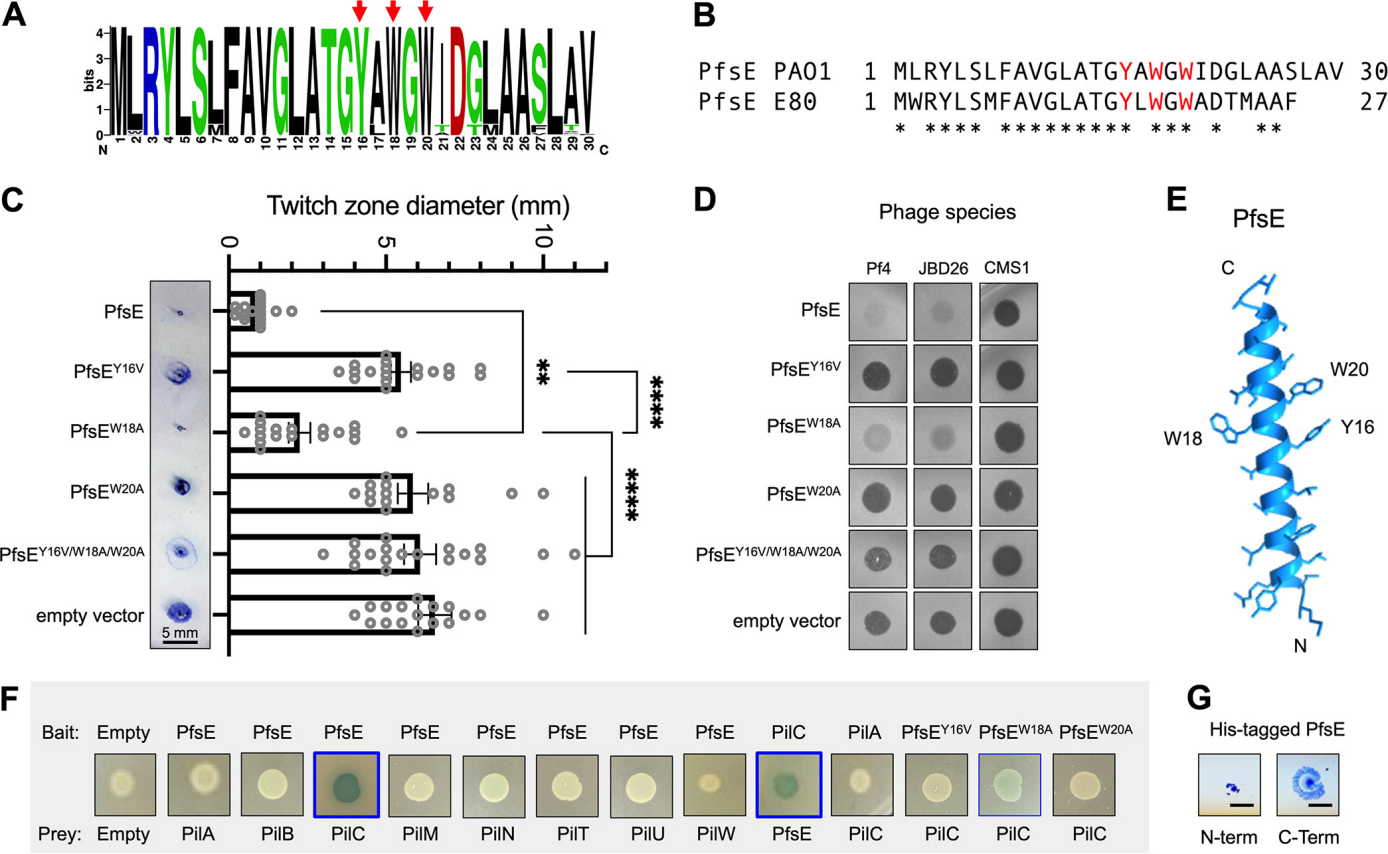
Conserved aromatic residues in PfsE are essential for the inhibition of type IV pili and resistance against Pf4 superinfection. (A) WebLogo (55) was used to construct a protein sequence logo for 312 PfsE sequences from Pf prophages infecting P. aeruginosa strains in the Pseudomonas genome database (24). Note that aromatic residues in the YXWGW motif (residues 16–20) are conserved (arrows). (B) There is 66% sequence identity (20/30) between two of the most highly diverged PfsE homologues found in Pf prophages residing in the P. aeruginosa PAO1 or E80 chromosomes. (C) Twitch motility was measured in PAO1^ΔPf4^ expressing wild-type or modified PfsE. Results are mean ± SEM of 16 experiments, unpaired Student's *t* test, **, *P* < 0.01, ****, *P* < 0.0001. (D) Lawns of PAO1^ΔPf4^ carrying the indicated expression vectors were induced with 0.01% arabinose and spotted with 3 μL of 10^9^ PFU/mL stocks of Pf4, JBD26 (pili-dependent phages), or CMS1 (a pili-independent phage). (E) AlphaFold (52) was used to predict the structure of PfsE. (F) A bacterial adenylate cyclase two-hybrid (BACTH) assay (25) was used to detect interactions between PfsE and the indicated T4P proteins. Interactions between bait and prey proteins are detected by β-galactosidase activity, as indicated by the production of blue pigment. Representative colonies are shown. (G) A 6×-His tag was added to the N- or C-terminus of PfsE, expressed in PAO1^ΔPf4^, and twitching was measured. Scale bar 5 mm.

The T4P complex consists of four subcomplexes: the outer membrane complex, an alignment complex that spans the periplasm, and two inner membrane complexes (23). Because PfsE is predicted to localize in the inner membrane (24), we hypothesized that PfsE would interact with inner membrane proteins of the T4P complex or proteins that interact with the T4P inner membrane complex. To test this hypothesis, we used a bacterial adenylate cyclase two-hybrid (BACTH) assay (25) to detect interactions between PfsE and the T4P proteins PilA, PilB, PilC, PilM, PilN, PilT, PilU, or PilW. In the BACTH assay, interactions between bait (PfsE) and prey (pilus proteins) are detected by β-galactosidase activity. High levels of β-galactosidase activity were observed only when PfsE was expressed with PilC (Fig. 6F), an inner membrane protein essential for T4P pilus biogenesis (26). Similar activity was observed when PilC was used as bait and PfsE as prey (Fig. 6F).

In an attempt to map residues that are important for the PsfE/PilC interaction, we examined the PfsE protein sequence and identified a conserved cluster of aromatic residues, YAWGW (Fig. 6A and B). As clusters of aromatic residues often facilitate protein-protein binding interactions (27, 28), we hypothesized that these residues may be required for suppression of twitching motility and resistance to T4P-dependent phages. To test this hypothesis, we introduced into PfsE the following point mutations: PfsE^Y16V^, PfsE^W18A^, PfsE^W20A^, and PfsE^Y16V/W18A/W20A^. These variant proteins were expressed in PAO1^ΔPf4^, and twitching motility and phage resistance were measured. PfsE^Y16V^, PfsE^W20A^, and PfsE^Y16V/W18A/W20A^ all lost the ability to suppress twitching motility (Fig. 6C) and did not promote resistance to the T4P-dependent phages Pf4 or JBD26 (Fig. 6D). PfsE^W18A^ partially suppressed both twitch motility and infection by T4P-dependent phages. When the PfsE^Y16V^ or PfsE^W20A^ point mutants were used as bait with PilC as the prey, β-galactosidase activity was not detected. The PfsE^W18A^ mutant showed some β-galactosidase activity in the BACTH assay, consistent with its intermediate twitch and phage resistance phenotypes. To determine if the loss of twitching and phage resistance was due to loss of the protein interaction or to instability of the protein, we used a Western blot to examine the steady-state levels of 6×-His-tagged wild-type PfsE, PfsE^Y16V^, and PfsE^W20A^ in E. coli. While we were able to detect wild-type PfsE using an anti-His antibody, we were unable to detect the PfsE^Y16V^ or PfsE^W20A^ point mutants (Fig. S3), suggesting that the loss of twitching motility, phage resistance, and the PilC interaction were due to low protein levels in the cell. These data suggest that Y16 and W20 play a role in stabilizing the PfsE protein fold or promoting its proper insertion into the membrane.

10.1128/mBio.02441-21.3 FIG S3 Wild-type PfsE protein is expressed in E. coli, but not the point mutants PfsE^Y16V^ or PfsE^W20A^. A His tag was cloned onto the N-terminus of wild-type or mutant PfsE in a pETDuet vector. Proteins were then expressed in E. coli BTH101. Protein was then run on SDS-PAGE, blotted, and detected using anti-His antibodies. A representative gel from triplicate experiments is shown. Download FIG S3, TIF file, 0.4 MB. Copyright © 2022 Schmidt et al. 2022 Schmidt et al. https://creativecommons.org/licenses/by/4.0/ This content is distributed under the terms of the Creative Commons Attribution 4.0 International license.

The binding of PfsE to the inner membrane protein PilC is consistent with the prediction that PfsE is itself an inner membrane protein. To determine the orientation that PfsE inserts itself into the inner membrane, we tagged the N- or C-terminus of PfsE with a poly-histidine (His) tag, which is positively charged and unable to insert into lipid membranes. Tagged PfsE was expressed in PAO1^ΔPf4^ and twitching motility measured. The N-terminal His tag had no impact on the ability of PfsE to inhibit twitching, while the C-terminal His tag prevented PfsE-mediated twitching inhibition (Fig. 6G). These results suggest that PfsE inserts into the inner membrane with the N-terminus oriented toward the cytoplasm.

**FIG 7:**
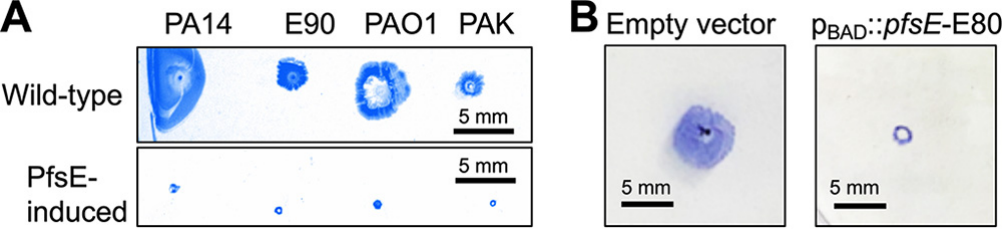
Divergent PfsE sequences inhibit twitching motility in various P. aeruginosa strains. (A) PfsE was expressed in *trans* in the indicated strains of P. aeruginosa and twitching motility measured after 24 h. Representative images are shown. (B) Twitching motility was assessed in PAO1**^Δ^**^Pf4^ carrying either the empty vector pHERD20T or p_BAD_-*pfsE-*E80 by standard twitch assay. Representative images are shown.

As PilC is highly conserved across many different strains of P. aeruginosa (26), we tested the ability of PfsE to inhibit twitching motility in P. aeruginosa strains PA14, PAK, and E90. We found that the PfsE sequence from Pf4 inhibited twitching in each of these strains (Fig. 7A). When the divergent PfsE sequence from P. aeruginosa strain E80 (see Fig. 6B) was expressed in P. aeruginosa PAO1^ΔPf4^, twitching motility was similarly inhibited (Fig. 7B). These results indicate a conserved mechanism of action.

## DISCUSSION

Many P. aeruginosa isolates are Pf lysogens (i.e., they harbor one or more Pf prophages in their chromosome) (1, 2). Lysogenized bacteria defend against infection by the same or similar phage through a mechanism called superinfection exclusion. For example, previously characterized superinfection exclusion mechanisms employed by P. aeruginosa phages include proteins Aqs1 from phage DMS3 (17) and Tip from phage D3112 (18), both of which inhibit T4P by binding to the T4P assembly ATPase PilB, which energizes pilus extension (26). This is the first report, to our knowledge, of a phage protein binding PilC to suppress T4P and prevent superinfection. Our data support a model where the Pf-encoded protein PfsE mediates superinfection exclusion by transiently suppressing T4P by binding to PilC to prevent pilus extension, which inhibits twitching motility and prevents infection by T4P-dependent phages. PfsE interactions with PilC may inhibit T4P either by blocking PilC from rotating, PilA loading, or by interfering with interactions between PilC and other pili proteins such as PilB.

To our knowledge, a transient superinfection exclusion phenotype has not previously been described for a phage. However, transient superinfection exclusion has been observed in animal viruses such as the RNA Pestivirus that causes bovine viral diarrhea (29). The transient nature of PfsE-mediated T4P inhibition may be related to highly stable PfsE-PilC binding interactions. If true, then if PfsE is downregulated as Pf re-enters the lysogenic replication life cycle, tight binding interactions between PfsE and PilC may titrate out PfsE as the cells divide, restoring T4P function. Alternatively, the conserved acidic residue D22 (see Fig. 6A) may pull the C-terminus of PfsE into the periplasmic space where PfsE could interact with periplasmic proteins that could disrupt PfsE-PilC binding (e.g., a periplasmic protease could degrade PfsE).

Our data suggest that PfsE inhibits pili extension. By inhibiting extension of the T4P cell surface receptor, PfsE may reduce the number of Pf virions that are “wasted” on nonproductive infections of an already infected host. This would allow Pf virions to accumulate in the environment, allowing Pf phage to spread and infect naive P. aeruginosa hosts. P. aeruginosa also benefits from the accumulation of filamentous Pf virions in the environment; as Pf virions accumulate in polymer-rich environments such as the biofilm matrix or host secretions (e.g., mucus), they spontaneously align, creating a large liquid crystalline lattice that protects P. aeruginosa from desiccation and some antibiotics (8, 30, 31). When encountered by immune cells, Pf virions induce a type I interferon antiviral response, which reduces the phagocytic uptake of bacteria by macrophages (4). Collectively, these phenotypes help explain why in P. aeruginosa PAO1 deleting the Pf4 prophage from the chromosome reduces bacterial virulence in murine lung (10) and wound (4) infection models.

PfsE inhibition of T4P may affect other bacterial behaviors. For example, in P. aeruginosa, T4P play important roles in virulence and biofilm formation (32, 33). T4P are critical virulence determinants in P. aeruginosa (33), and inhibition of T4P by PfsE could affect P. aeruginosa virulence potential. This possibility is consistent with our previous work demonstrating that Pf4 superinfection promotes a noninvasive infection phenotype *in vivo* (5). Under some conditions such as nutrient limitation, suppression of T4P is thought to contribute to biofilm dispersion (34). Pf4 superinfection contributes to the biofilm life cycle as well by inducing cell death and lysis, which produces the characteristic voids in the center of mature microcolonies of biofilms grown in flow cells (10–12). In dispersed cell populations, Pf4 gene expression is upregulated while T4P genes are downregulated (34, 35). Thus, it is possible that in response to Pf4 superinfection, PfsE expression contributes to biofilm dispersal by suppressing T4P.

Phage therapy holds great potential in combating multidrug-resistant bacterial infections in several settings (36–41). Unfortunately, bacteria can develop resistance to therapeutic phages, causing treatment failure ((42) and references therein). In some cases, heritable phage resistance mutations cannot account for phage therapy failure as bacteria remain sensitive to phage infection *ex vivo* (42–45). Because Pf prophages are prevalent among P. aeruginosa clinical isolates and PfsE is encoded by all Pf lysogens, it is possible that PfsE could cause some phage therapies to fail. Conversely, PfsE could be leveraged as a therapeutic. Recent work demonstrates that the Tip protein from phage D3112 inhibits T4P by blocking the activity of PilB (18). A peptide mimic of the Tip protein inhibits T4P *in vitro*, and when given topically to P. aeruginosa, the peptide reduced virulence in a *Drosophila* infection model (46). This approach could potentially be adapted to PfsE by synthesizing a peptide that contains the aromatic amino acid motif YAWGW.

## MATERIALS AND METHODS

### Bacterial strains, plasmids, and growth conditions.

Strains, plasmids, and their sources are listed in Table 1, and primers are listed in Table 2. Unless indicated otherwise, bacteria were grown in lysogeny broth (LB) at 37°C with shaking and supplemented with antibiotics (Sigma) or 0.1% IPTG when appropriate. Unless otherwise noted, antibiotics were used at the following concentrations: gentamicin (10 or 30 μg mL^−1^), ampicillin (100 μg mL^−1^), kanamycin (50 μg mL^−1^), carbenicillin (50 μg mL^−1^).

**TABLE 1:** Bacterial strains, phage, and plasmids used in this study

Strain	Description	Source
Escherichia coli		
DH5α	New England Biolabs	(15)
S17	λpir-positive strain	(15)
BTH101	BACTH reporter strain	(25)
P. aeruginosa		
PAO1	Wild type	(56)
PAO1 Δ*pilA*	Clean deletion of *pilA* from PAO1	(57)
PAO1 Δ*intF4*	Clean deletion of *intF4* from PAO1	(8)
PAO1 Δ*intF4/pfsE*	Clean deletion of *pfsE* from PAO1 Δ*intF4*	This study
PAO1 ΔPf4	Clean deletion of the Pf4 prophage from PAO1	This study
PA14	Wild type	(58)
PAK	Wild type	ATCC 25102
E90	Clinical CF P. aeruginosa isolate	(59)
Bacteriophage strains		
Pf4	Inoviridae	(8)
JBD26	Siphoviridae	(20)
CMS1	Podoviridae	This study
DMS3	Siphoviridae	(60)
OMK01	Myoviridae	(61)
LPS-5	Podoviridae	Felix Biotech
Plasmids		
pHERD20T	AmpR, expression vector with araC-P_BAD_ promoter	(49)
pHERD30T	GmR, expression vector with araC-P_BAD_ promoter	(49)
pHERD30T-*PA0717*	pBAD::*PA0717*	(19)
pHERD30T-*PA0718*	pBAD::*PA0718*	(19)
pHERD30T-*PA0719*	pBAD::*PA0719*	This study
pHERD30T-*PA0720*	pBAD::*PA0720*	(19)
pHERD30T-*pfsE*	pBAD::*pfsE*	(19)
pHERD30T-*PA0722*	pBAD::*PA0722*	This study
pHERD30T-*PA0723*	pBAD::*PA0723*	This study
pHERD30T-*PA0724*	pBAD::*PA0724*	This study
pHERD30T-*PA0725*	pBAD::*PA0725*	(19)
pHERD30T-*PA0726*	pBAD::*PA0726*	This study
pHERD30T-*PA0727*	pBAD::*PA0727*	This study
pHERD30T-*intF4*	pBAD::*intF4*	This study
pHERD20T-*xisF4*	pBAD::*xisF4*	(9)
pKT25	BACTH construct	(25)
pUT18C	BACTH construct	(25)
pHERD20T-*pfsE*	pBAD::*pfsE*	This study
pHERD20T-*pfsE* ^Y16V^	pBAD:: *pfsE* ^Y16V^	This study
pHERD20T-*pfsE* ^W18A^	pBAD:: *pfsE* ^W18A^	This study
pHERD20T-*pfsE* ^W20A^	pBAD:: *pfsE* ^W20A^	This study
pHERD20T-*pfsE*^Y16A/W18A/W20A^	pBAD:: *pfsE* ^Y16A/W18A/W20A^	This study

**TABLE 2:** Primers used in this study

Purpose/name	Sequence (5′–3′)
Cloning	
*PfsE_p18CFwd*	TACGTCTAGAGCTCCGCTATCTCTCGCTGTTCGCGGTAGG
*PfsE_p18CRev*	TACGGGTACCTCAAACAGTCAGGGAGGCCGCTAGG
*PfsE_Y16VFwd*	CTGGCCACCGGCGTGGCCTGG
*PfsE_Y16VRev*	CCAGCCCCAGGCCACGCCGGT
*PfsE_W18AFwd*	ACCGGCTACGCCGCCGGCTGG
*PfsE_W18ARev*	TCGATCCAGCCGGCGGCGTAGC
*PfsE_W20AFwd*	TACGCCTGGGGCGCCATCGACG
*PfsE_W20ARev*	CTAGGCCGTCGATGGCGCCCCAGG
*ΔpfiT* primers	
*attB1-pfiT-UpF*	ggggataagtttgtacaaaaaagcaggcttcTTCAACCCGCTCATAGGTT
*pfiT-UpR*	TCAGGAGTAGAAAGCCATCACATTAAACCTCCTTATTCTGG
*PfiT-DownF*	TGATGGCTTTCTACTCCTGA
*attB2-PfiT-DownR*	ggggaccactttgtacaagaaagctgggtaAGCCGCTCAACCCGATCTA
*PfiT seq F*	CCACACGTTCGCCAGTCACTT
*PfiT seq R*	AATGCCGGCCACTTCATCGAC
*ΔPf4* primers	
*Pf4-UpF-GWL*	tacaaaaaagcaggctTCTGGGAATACGACGGGGGC
*Pf4-UpR-GM*	tcagagcgcttttgaagctaattcgGATCCCAATGCAAAAGCCCC
*Pf4-DnF-GM*	aggaacttcaagatccccaattcgCGTCATGAGCTTGGGAAGCT
*Pf4-DnR-GWR*	tacaagaaagctgggtTGGCAGCAGACCCAGGACGC
*pf4-out F*	AGTGGCGGTTATCGGATGAC
*pf4-out R*	TCATTGGGAGGCGCTTTCAT

### Construction of strain ΔPf4.

The Pf4 prophage was deleted from the PAO1 chromosome by allelic exchange (47), producing a clean and unmarked ΔPf4 deletion with the Pf4 att site intact. All primers used for strain construction are given in Table 2. The Pf4 prophage contains a toxin-antitoxin (TA) pair (48). The presence of the Pf4-encoded PfiTA system likely explains the low efficiency at which the Pf4 prophage can be deleted from the PAO1 chromosome (10); deletion of the Pf4 prophage results in loss of the antitoxin gene *pfiA*, and cells without the antitoxin are killed by the longer-lived toxin PfiT (48). Thus, the *pfiT* toxin gene was first deleted from PAO1 by allelic exchange (47). Briefly, the upstream region of *pfIT* (*pfiT’*) and the downstream region of *pfIT* (*‘pfiT*) were amplified using the primer pairs attB1-pfiT-UpF/pfiT-UpR and PfiT-DownF/attB2-PfiT-DownR, respectively (Table 2). These were then assembled using SOE-PCR. The resulting fragment was cloned into pENTRpEX18-Gm, transformed into Escherichia coli S17λ*pir*, and subsequently mobilized into P. aeruginosa PAO1 via biparental mating. Merodiploid P. aeruginosa was selected on Vogel-Bonner minimal medium (VBMM) agar containing 60 μg ml^−1^ gentamicin, followed by recovery of deletion mutants on no-salt LB (NSLB) medium containing 10% sucrose. Candidate mutants were confirmed by PCR and sequencing using primer pair PfiT seq F/PfiT seq R. The remaining Pf4 prophage was then deleted from Δ*pfiT* using the same allelic exchange strategy described for Δ*pfiT* using primers Pf4-UpF-GWL, Pf4-UpR-GM, Pf4-DnF-GM, and Pf4-DnR-GWR (Table 2), producing an unmarked clean deletion of Pf4 with an intact att site. Candidate mutants were confirmed by PCR using primer pair pf4-out F/pf4-out R and sequence confirmed. Supernatants collected from overnight cultures of this ΔPf4 strain did not produce detectable plaques on lawns of PAO1 or ΔPf4, and the ΔPf4 genotype was routinely PCR confirmed prior to experiments using this strain to avoid reinfection by exogenous Pf4 virions in the laboratory environment.

### Phage expression constructs.

The indicated Pf4 genes or *pfsE* point mutant genes were cloned into the arabinose-inducible expression constructs pHERD20T or pHERD30T (49), were obtained from reference (19), or were made by Genewiz (Table 1). Final constructs were all sequence verified.

### Twitch motility assays.

Twitching motility was assessed by stab inoculating the indicated strains through a 1.5% LB agar plate to the underlying plastic dish. Agar was supplemented with 0.1% arabinose or antibiotics when appropriate. After incubation for 24 h, the agar was carefully removed, and the zone of motility on the plastic dish was visualized and measured after staining with 0.05% Coomassie brilliant blue, as previously described (50). Twitch zones were measured by placing the plastic dish onto a ruler and imaging with a Bio-Rad GelDoc GO imaging system using preset parameters for Coomassie-stained gels.

### Plaque assays.

Plaque assays were performed using ΔPf4 or isogenic PAO1 as indicator strains grown on LB plates. Phages in filtered supernatants were serially diluted 10× in PBS and spotted onto lawns of the indicated indicator strain. Plaques were imaged after 18 h of growth at 37°C.

### Pf4 phage virion quantitation by qPCR.

Pf4 virion copy number was measured using qPCR as previously described (22). Briefly, filtered supernatants were treated with DNase I (10 μL of a 10 mg/mL stock per mL supernatant) followed by incubation at 70°C for 10 min to inactivate the DNase. Ten μL reaction volumes contained 5 μL SYBR Select Master Mix (Life Technologies, Grand Island, NY), 100 nM primer attR-F and attL-R (Table 2), and 2 μL supernatant. Primers attR-F and attL-R amplify the recircularization sequence of the Pf4 replicative form and thus do not amplify linear Pf4 prophage sequences that may be present in contaminating chromosomal DNA. Cycling conditions were as follows: 50°C 2 min, 95°C 2 min, (95°C, 15 sec, 60°C 1 min) × 40 cycles. A standard curve was constructed using plasmids containing the template sequence at a known copy number per milliliter. Pf4 copy numbers were then calculated by fitting Ct values of the unknown samples to the standard curve.

### Pf4 virion induction.

P. aeruginosa strains PAO1, Δ*intF4*, and Δ*intF4/pfsE* were made competent by 300 mM sucrose washes (51) and electroporated with the arabinose-inducible expression vectors pHERD20T-*xisF4* and pHERD30T-*intF4.* Double transformants were grown in LB supplemented with gentamicin and carbenicillin to an OD_600_ of 0.3 and induced with 0.1% arabinose. Bacteria were grown for 18 h, pelleted by centrifugation, and supernatants were filtered through a 0.22 μm filter (Millipore Millex GP) followed by DNase treatment. Pf4 virion titers were measured by qPCR, as described above. Pf4 copy numbers in each supernatant were normalized to the same titer by diluting with PBS.

### Growth curves.

Overnight cultures were diluted to an OD_600_ of 0.05 in 96-well plates containing LB and, if necessary, the appropriate antibiotics. Over the course of 24 h, OD_600_ was measured in a CLARIOstar (BMG Labtech) plate reader at 37C with shaking prior to each measurement.

### Transmission electron microscopy.

Cells were grown to midlog (OD_600_ 0.4), washed with PBS, fixed with 4% formamide, and placed on a grid and negatively stained with uranyl acetate. Cells were imaged on a Hitachi H-7800 120 kV TEM.

### Bacterial two-hybrid assays.

Bacterial two-hybrid assays were performed as described previously (25). PfsE was cloned into plasmid constructs (pKT25, pUT18C) using relevant primers (Table 2). Escherichia coli BTH101 cells were cotransformed with plasmid constructs containing different sets of genes. Three independent colonies were grown overnight at 30°C in LB media containing the appropriate selection, 2 μL of each was plated onto X-gal, and MacConkey agar plates containing the appropriate selection and 1 mM IPTG and incubated at 30°C for 48 h. A color change on both plates indicates an interaction between the genes encoded in the plasmids.

### PfsE modeling.

AlphaFold (52) was used to predict the secondary structure of PfsE. The .pdb file was downloaded for PfsE “model one” and visualized using UCSF ChimeraX (53).

### Statistical analyses.

Differences between data sets were evaluated by an unpaired Student's *t* test, using GraphPad Prism version 5.0 (GraphPad Software, San Diego, CA). *P* values of < 0.05 were considered statistically significant.
