# Supplementary figures and images for: A Filamentous Bacteriophage Protein Inhibits Type IV Pili To Prevent Superinfection of Pseudomonas aeruginosa
Source: mBio. 2022 Jan 18;13(1):e02441-21. doi: 10.1128/mbio.02441-21 (PMC8764522; doi:10.1128/mbio.02441-21)

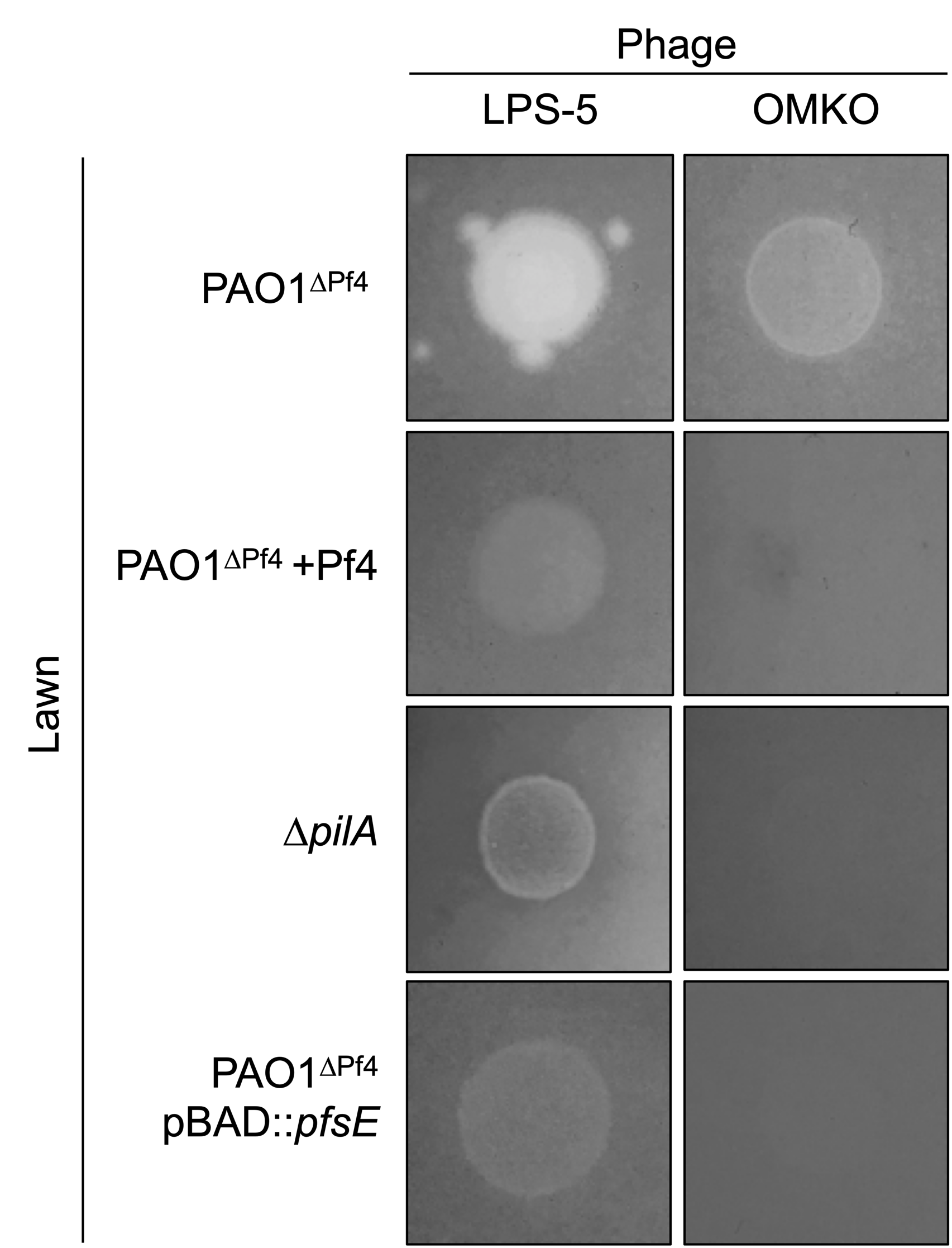

Supplement: FIG S1 [file mbio.02441-21-sf001.tif]

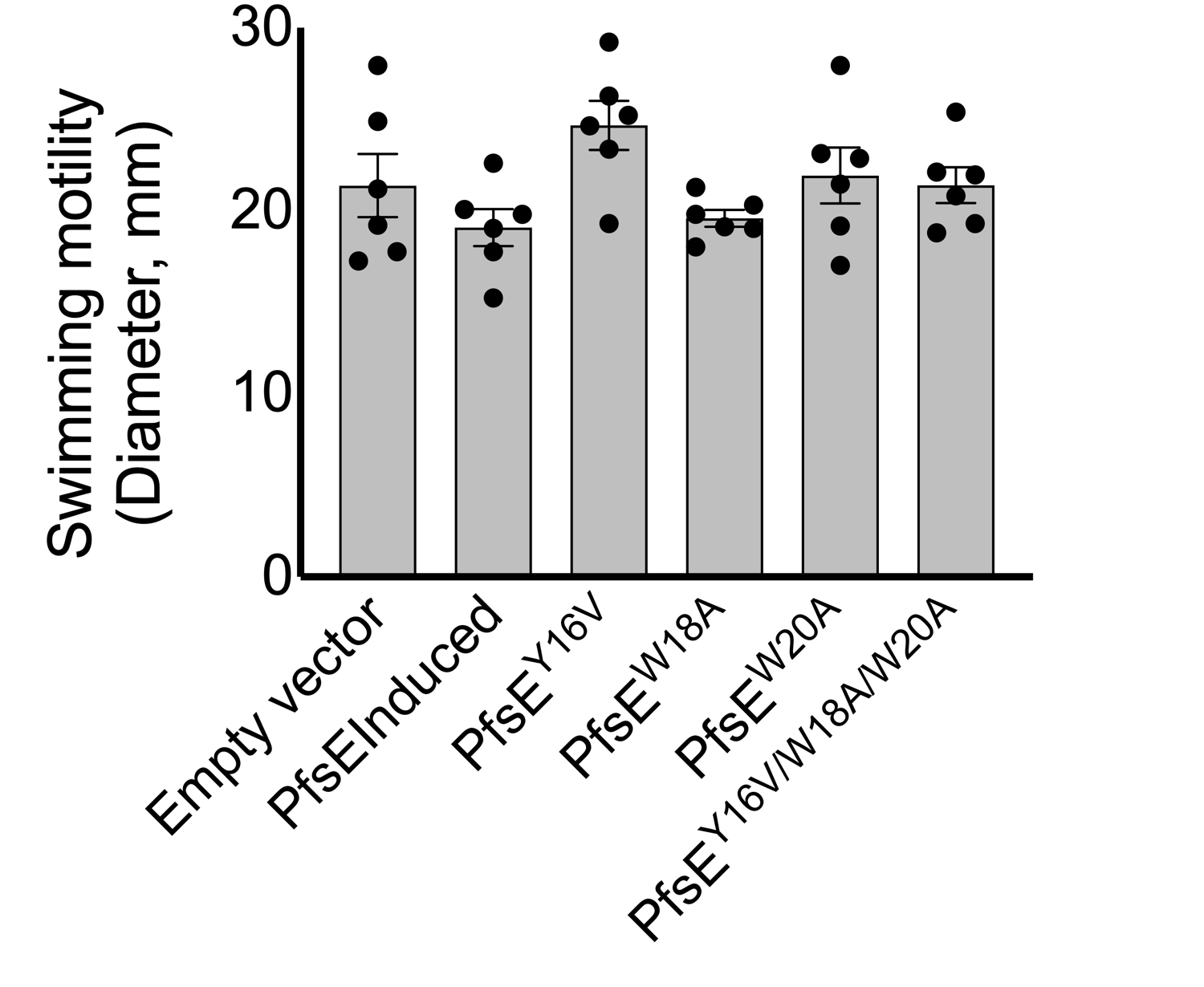

Supplement: FIG S2 [file mbio.02441-21-sf002.tif]

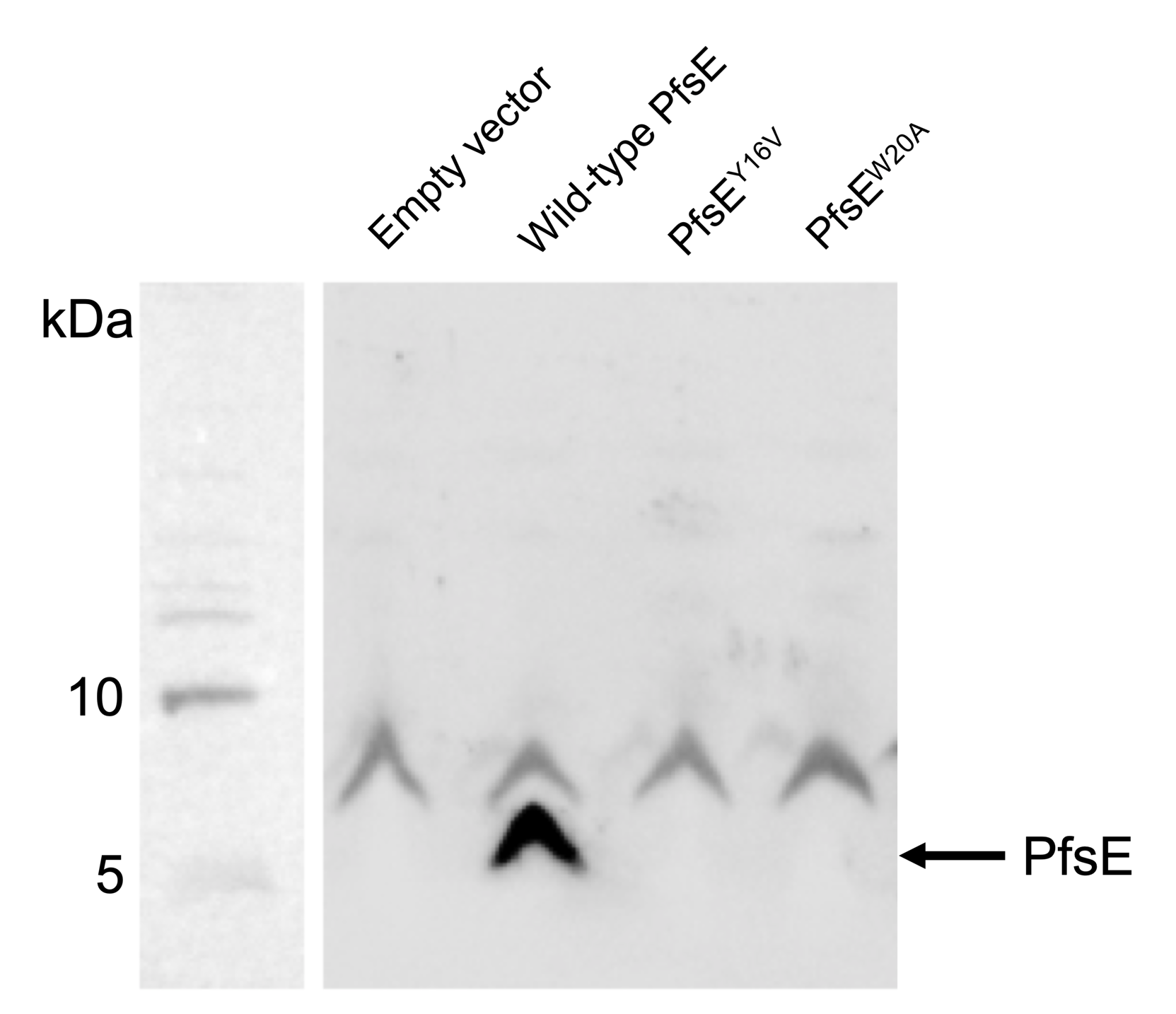

Supplement: FIG S3 [file mbio.02441-21-sf003.tif]
